# The diagnostic role of the systemic inflammation index in patients with immunological diseases: a systematic review and meta-analysis

**DOI:** 10.1007/s10238-024-01294-3

**Published:** 2024-01-29

**Authors:** Arduino A. Mangoni, Angelo Zinellu

**Affiliations:** 1 Discipline of Clinical Pharmacology, College of Medicine and Public Health, Flinders University and Flinders Medical Centre, Bedford ParkAdelaide, SA 5042 Australia; 2 Department of Clinical Pharmacology, Flinders Medical Centre, Southern Adelaide Local Health Network, Adelaide, Australia; 3 Department of Biomedical Sciences, University of Sassari, Sassari, Italy

**Keywords:** Systemic inflammation index, SII, Immunological diseases, Autoinflammatory diseases, Mixed-pattern diseases, Diagnosis, Biomarkers, Active disease, Remission

## Abstract

The identification of novel, easily measurable biomarkers of inflammation might enhance the diagnosis and management of immunological diseases (IDs). We conducted a systematic review and meta-analysis to investigate an emerging biomarker derived from the full blood count, the systemic inflammation index (SII), in patients with IDs and healthy controls. We searched Scopus, PubMed, and Web of Science from inception to 12 December 2023 for relevant articles and evaluated the risk of bias and the certainty of evidence using the Joanna Briggs Checklist and the Grades of Recommendation, Assessment, Development, and Evaluation Working Group system, respectively. In 16 eligible studies, patients with IDs had a significantly higher SII when compared to controls (standard mean difference, SMD = 1.08, 95% CI 0.75 to 1.41, *p* < 0.001; I^2^ = 96.2%, *p* < 0.001; moderate certainty of evidence). The pooled area under the curve (AUC) for diagnostic accuracy was 0.85 (95% CI 0.82–0.88). In subgroup analysis, the effect size was significant across different types of ID, barring systemic lupus erythematosus (*p* = 0.20). In further analyses, the SII was significantly higher in ID patients with active disease vs. those in remission (SMD = 0.81, 95% CI 0.34–1.27, *p* < 0.001; I^2^ = 93.6%, *p* < 0.001; moderate certainty of evidence). The pooled AUC was 0.74 (95% CI 0.70–0.78). Our study suggests that the SII can effectively discriminate between subjects with and without IDs and between ID patients with and without active disease. Prospective studies are warranted to determine whether the SII can enhance the diagnosis of IDs in routine practice. (PROSPERO registration number: CRD42023493142).

**Supplementary Information:**

The online version contains supplementary material available at 10.1007/s10238-024-01294-3.

## Introduction

The term “immunological diseases (IDs)” has been introduced over the last 20 years to describe a wide range of chronic conditions characterized by a self-directed tissue inflammation process that is not necessarily associated with alterations in the function of B and T cells, the hallmark of conventional autoimmune disorders [1–6]. As a result, IDs consist of an autoinflammatory-autoimmune continuum that includes monogenic (e.g., Familial Mediterranean Fever) and polygenic (e.g., Crohn’s disease, ulcerative colitis, UC, gout, and giant cell arteritis) autoinflammatory diseases, mixed-pattern diseases (e.g., ankylosing spondylitis, AS, psoriasis, and Bechet’s disease), and monogenic (e.g., autoimmune lymphoproliferative syndrome) and polygenic (e.g., rheumatoid arthritis, RA, Addison’s disease, systemic lupus erythematosus, SLE, and dermatomyositis) autoimmune diseases [1, 7, 8].

The robust evidence of dysregulation of inflammatory pathways in IDs has led to the routine use of circulating biomarkers of inflammation, e.g., C-reactive protein (CRP), erythrocyte sedimentation rate (ESR), and ferritin, to diagnose the presence of specific IDs and/or a state of active disease vs. remission in clinical practice [9–13]. However, their limited diagnostic accuracy in several types of IDs has stimulated a significant body of research to identify better biomarkers [9, 14–16]. In this context, alterations in the count and ratios of specific blood cell types, e.g., neutrophils, platelets, and lymphocytes, have been studied to diagnose the presence of IDs and predict disease progression [17–23]. Over the last decade, another hematological cell index, the systemic inflammation index [SII = (neutrophil count x platelet count)/lymphocyte count] has been investigated in patients with cancer [24, 25], cardiovascular disease [26], liver disease [27], and, more recently, in patients with coronavirus disease 2019 (COVID-19) [28]. Notably, in studies of COVID-19 the SII has shown a superior predictive capacity for adverse clinical outcomes when compared to other hematological indexes, e.g., the neutrophil-to-lymphocyte ratio [29].

Given the increasing interest in the potential clinical utility of the SII, we conducted a systematic review and meta-analysis of studies investigating this hematological index in patients with IDs and healthy controls and in ID patients with active disease and remission. We speculated that the presence of IDs was associated with significantly higher SII values vs. healthy controls and that the presence of active disease in patients with IDs was associated with higher SII values vs. patients in remission. We also investigated the presence of possible associations between the effect size of the between-group differences in SII values and several relevant demographic and clinical parameters, including specific IDs, ID duration, CRP, and ESR.

## Materials and methods

### Search strategy and study selection

We conducted a systematic search for articles in the electronic databases PubMed, Web of Science, and Scopus from their inception to 05 December 2023 according to the following terms and their combinations capturing the conditions listed in published classifications of IDs [1, 7, 8]: “systemic immune-inflammation index” OR “SII” AND “immunological diseases” OR “rheumatoid arthritis” OR “psoriatic arthritis” OR “reactive arthritis” OR “ankylosing spondylitis” OR “systemic lupus erythematosus” OR “systemic sclerosis” OR “scleroderma” OR “Sjogren’s syndrome” OR “vasculitis” OR “Behçet’s disease” OR “connective tissue diseases” OR “idiopathic inflammatory myositis” OR “polymyositis” OR “dermatomyositis” OR “gout” OR “pseudogout” OR”systemic vasculitis” OR “ANCA-associated vasculitis” OR “Takayasu arteritis” OR “polyarteritis nodosa” OR “osteoarthritis” OR “fibromyalgia” OR”Crohn’s disease” OR “ulcerative colitis” OR “granulomatous polyangiitis” OR”Henoch-Schönlein purpura” OR “Wegener’s granulomatosis” OR “uveitis” OR “type 1 diabetes” OR “coeliac disease” OR “myasthenia gravis" OR “pemphigus” OR “Addison’s disease” OR “Goodpasture syndrome” OR “autoimmune thyroid disease” OR “primary biliary cirrhosis” OR “autoimmune gastritis” OR “erythema nodosum” OR “sarcoidosis”.

Two independent investigators screened each abstract and, if relevant, the full-text article according to the following inclusion criteria: (i) assessment of the SII, (ii) comparisons between patients with IDs and healthy controls (case–control design), (iii) age ≥ 18 years, (iv) English language, and (v) full-text available. The references of each article were hand searched for additional studies.

The following information was independently extracted from each article and transferred to an electronic spreadsheet for analysis: year of publication, first author, study design, study country, type of ID, disease duration, sample size, age, male to female ratio, markers of inflammation (erythrocyte sedimentation rate, ESR, and C-reactive protein, CRP), the area under the receiver operating characteristic curve (AUROC) with 95% confidence intervals (CIs), and diagnostic sensitivity and specificity for the presence of ID and active disease.

We assessed the risk of bias of each study using the items listed in the Joanna Briggs Institute Critical Appraisal Checklist for analytical cross-sectional studies [30]. Studies addressing ≥ 75, ≥ 50 and < 75%, and < 50% of the checklist items were ranked as having a low, intermediate, or high risk of bias, respectively. The certainty of evidence was assessed using the Grades of Recommendation, Assessment, Development and Evaluation (GRADE) Working Group system which considers the study design (retrospective or prospective), the risk of bias, the presence of unexplained heterogeneity, the indirectness of evidence, the imprecision of the results, the effect size (small, SMD < 0.5, moderate, SMD 0.5–0.8, and large, SMD > 0.8) [31], and the probability of publication bias [32]. We complied with the Preferred Reporting Items for Systematic reviews and Meta-Analyses (PRISMA) 2020 statement (Supplementary Table 1 and 2) [33], and registered the study protocol in the International Prospective Register of Systematic Reviews (PROSPERO registration number: CRD42023493142).

### Statistical analysis

Between-group differences in SII values were assessed by creating forest plots of standardized mean differences (SMDs) and 95% CIs. A *p* value < 0.05 was considered statistically significant. Appropriate methods were used to extrapolate the means and standard deviations from the medians and interquartile ranges or ranges [34]. The heterogeneity of the SMD across different studies was assessed using the Q-statistic (significance level set at a *p* value < 0.10) and ranked as low (I^2^ ≤ 25%), moderate (25% < I^2^ < 75%), or high (I^2^ ≥ 75%) [35, 36]. A random-effect model based on the inverse-variance method was used in the presence of high heterogeneity. Sensitivity analysis was conducted to assess the stability of the results of the meta-analysis [37].

The presence of publication bias was assessed using the Begg’s and Egger’s tests and the “trim-and-fill” method [38–40]. The midas command was used to assess the diagnostic performance of the SII for the presence of IDs and/or active disease by estimating the summary receiver operating characteristic (SROC) [41]. True positive (TP), false positive (FP), false negative (FN), and true negative (TN) values were either directly extracted or calculated from individual articles.

Univariate meta-regression and subgroup analyses were conducted to investigate possible associations between the SMD and the year of publication, study design, study country, ID type and duration, sample size, age, male to female ratio, ESR, and CRP. All statistical analyses were performed using Stata 14 (Stata Corp., College Station, TX, USA).

## Results

### Study selection

**Fig. 1:**
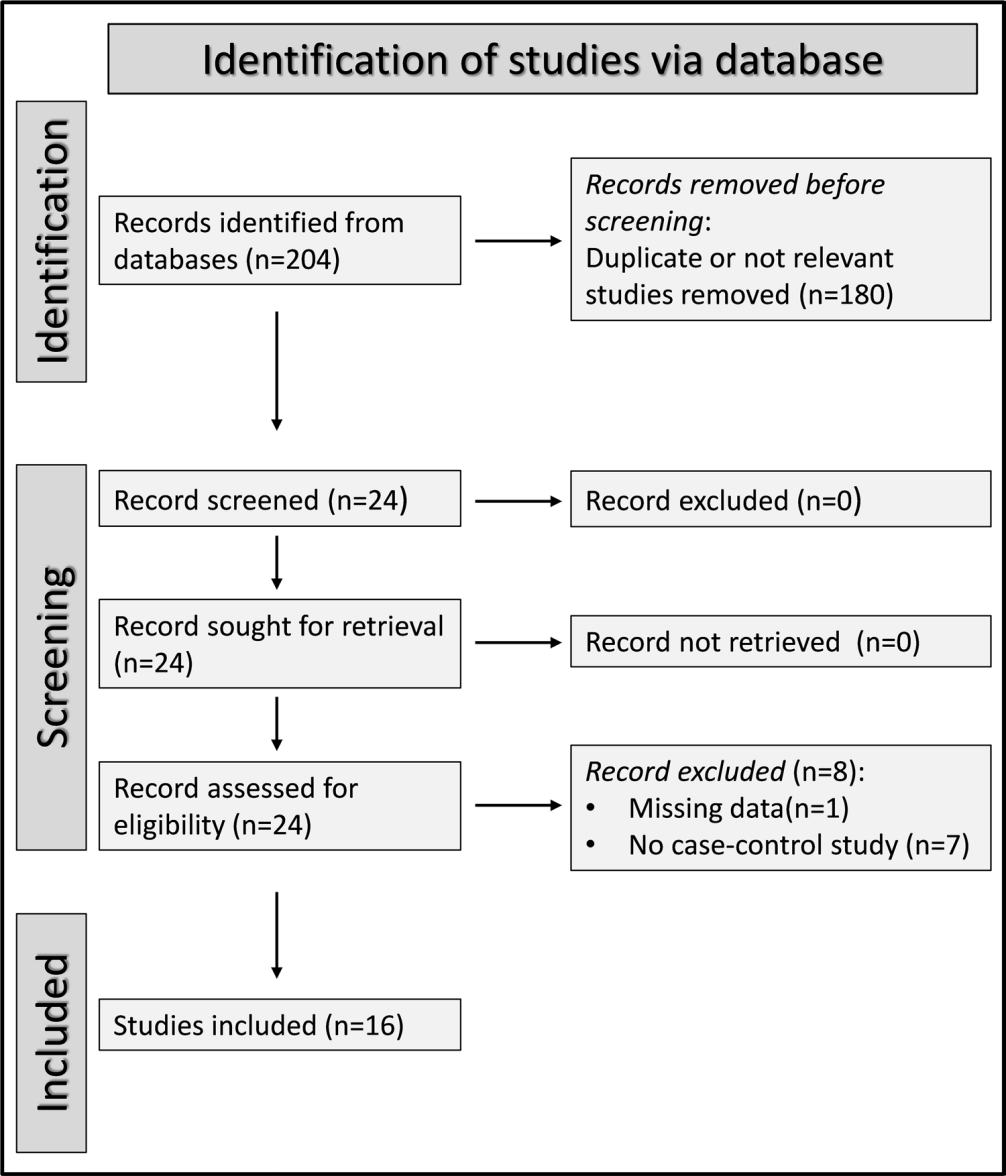
PRISMA 2020 flow diagram

After initially identifying a total of 204 articles, 180 were excluded because they were either duplicates or irrelevant. Following a full-text assessment of the remaining 24 articles, one study was excluded because it did not report relevant information and other seven were excluded because they did not have a case–control design. Therefore, 16 studies published between 2021 and 2023 were included in the final analysis [42–57] (Fig. 1 and Table 1). The initial level of certainty was rated as low (rating 2) given the cross-sectional design of all studies.

**Table 1:** Studies investigating the systemic inflammation index in patients with immunological diseases and healthy controls

Study	Healthy controls				Patients with immunological diseases				Disease	Study design
	n	Age (years)	M/F	SII (mean ± SD)	n	Age (years)	M/F	SII (mean ± SD)		
Kurtul BE et al. [42]	46	41	24/22	438 ± 122	46	41	22/24	680 ± 312	Uveitis	R
Satis S et al. [43]	31	47	11/20	597 ± 58	109	50	24/85	666 ± 33	RA	R
Wu J et al. [44]	63	34	48/15	297 ± 110	136	33	118/18	492 ± 246	AS	R
Xie Y et al., 2021, China [45]	185	42	113/72	344 ± 36	187	42	119/68	637 ± 139	UC	R
Zhang MH et al. [46]	172	48	96/76	402 ± 69	172	48	91/81	1126 ± 301	UC	R
Choe JY et al. [47]	80	58	5/75	387 ± 227	123	56	15/108	968 ± 591	RA	R
Kelesoglu Dincer AB et al. [48]	103	63	40/63	468 ± 188	106	59	47/59	616 ± 390	PsA	R
Luo Q et al. [49]	75	33	55/20	413 ± 204	79	32	55/24	874 ± 781	AS	P
Taha SI et al. (a), [50]	100	73	49/51	510 ± 221	100	67	19/81	733 ± 493	RA	P
Taha SI et al. (b), [50]	100	73	49/51	510 ± 221	100	65	16/84	537 ± 474	SLE	P
Taha SI et al. (c), [50]	100	73	49/51	510 ± 221	50	68	33/17	838 ± 408	AS	P
Choe JY et al. [51]	71	60	0/71	409 ± 277	257	61	0/257	697 ± 579	RA	R
Jiang Y et al. (a), 2023, China [52]	194	42	194/0	349 ± 137	474	43	474/0	572 ± 314	Gout	R
Jiang Y et al. (b), [52]	194	42	194/0	349 ± 137	399	42	399/0	426 ± 185	Gout	R
Karadeniz H et al. (a), [53]	27	43	12/15	484 ± 182	30	55	17/13	1707 ± 1343	IgG4-RD	P
Karadeniz H et al. (b), [53]	27	43	12/15	484 ± 182	46	54	14/32	2259 ± 1556	Sarcoidosis	P
Karadeniz H et al. (c), [53]	27	43	12/15	484 ± 182	38	48	16/22	2533 ± 1780	GPA	P
Ozdemir A et al. [54]	76	33	10/66	457 ± 579	76	33	11/65	1159 ± 1834	SLE	R
Sariyildiz A et al. [55]	50	45	33/17	418 ± 165	100	45	68/32	603 ± 288	AS	P
Tarabeih N et al. [56]	519	52	251/268	455 ± 314	98	56	30/68	615 ± 406	OA	P
Yan J et al. [57]	106	43	62/44	449 ± 233	167	43	94/83	1159 ± 861	UC	R

*AS* ankylosing spondylitis, *GPA* granulomatosis polyangiitis, *IgG4-RD* IgG4-related disease, *M/F* male to female ratio, *OA* osteoarthritis, *P* prospective, *PsA* psoriatic arthritis, *R* retrospective, *RA* rheumatoid arthritis, *SD* standard deviation, *SII* systemic inflammation index, *SLE* systemic lupus erythematosus, *UC* ulcerative colitis

### SII in patients with immunological diseases and healthy controls

We identified 16 studies reporting 21 group comparisons which investigated a total of 2893 patients with IDs (mean age 48 years, 42% females) and 2346 healthy controls (mean age 50 years, 44% females) [42–57] (Table 1). Six studies were conducted in China [44–46, 49, 52, 57], six in Turkey [42, 43, 48, 53–55], two in South Korea [47, 51], one in Egypt [50], and one in Israel [56]. Four group comparisons included patients with RA [43, 47, 50, 51], four with AS [44, 49, 50, 56], three with UC [45, 46, 57], two with gout [52], two with SLE [50, 54], one with psoriatic arthritis (PsA) [48], one with OA [56], one with uveitis [42], one with sarcoidosis [53], one with granulomatous polyangiitis (GPA) [53], and one with IgG4-related disease (IgG4-RD) [53]. The study design was retrospective in 11 studies [42–48, 51, 52, 54, 57], and prospective in the remaining five [49, 50, 53–56]. The risk of bias was assessed as low in 13 studies [42, 44–47, 49–56], and moderate in the remaining three [43, 48, 57] (Table 2).

**Table 2:** Assessment of the risk of bias using the Joanna Briggs Institute critical appraisal checklist

Study	Were the inclusion criteria clearly defined?	Were the subjects and the setting described in detail?	Was the exposure measured in a reliable way?	Were standard criteria used to assess the condition?	Were confounding factors identified?	Were strategies to deal with confounding factors stated?	Were the outcomes measured in a reliable way?	Was appropriate statistical analysis used?	Risk of bias
Kurtul BE et al. [42]	Yes	Yes	Yes	Yes	No	No	Yes	Yes	Low
Satis S et al. [43]	No	No	Yes	Yes	No	No	Yes	Yes	Moderate
Wu J et al. [44]	Yes	Yes	Yes	Yes	Yes	Yes	Yes	Yes	Low
Xie Y et al. [45]	Yes	Yes	Yes	Yes	Yes	Yes	Yes	Yes	Low
Zhang MH et al. [46]	Yes	Yes	Yes	Yes	Yes	Yes	Yes	Yes	Low
Choe JY et al. [47]	Yes	Yes	Yes	Yes	Yes	Yes	Yes	Yes	Low
Kelesoglu Dincer AB et al. [48]	No	No	Yes	Yes	No	No	Yes	Yes	Moderate
Luo Q et al. [49]	Yes	Yes	Yes	Yes	Yes	Yes	Yes	Yes	Low
Taha SI et al. [50]	Yes	Yes	Yes	Yes	Yes	Yes	Yes	Yes	Low
Choe JY et al. [51]	Yes	Yes	Yes	Yes	No	No	Yes	Yes	Low
Jiang Y et al. [52]	Yes	Yes	Yes	Yes	Yes	Yes	Yes	Yes	Low
Karadeniz H et al. [53]	Yes	Yes	Yes	Yes	No	No	Yes	Yes	Low
Ozdemir A et al. [54]	Yes	Yes	Yes	Yes	No	No	Yes	Yes	Low
Sariyildiz A et al. [55]	Yes	Yes	Yes	Yes	No	No	Yes	Yes	Low
Tarabeih N et al. [56]	Yes	Yes	Yes	Yes	Yes	Yes	Yes	Yes	Low
Yan J et al. [57]	No	Yes	Yes	Yes	No	No	Yes	Yes	Moderate

**Fig. 2:**
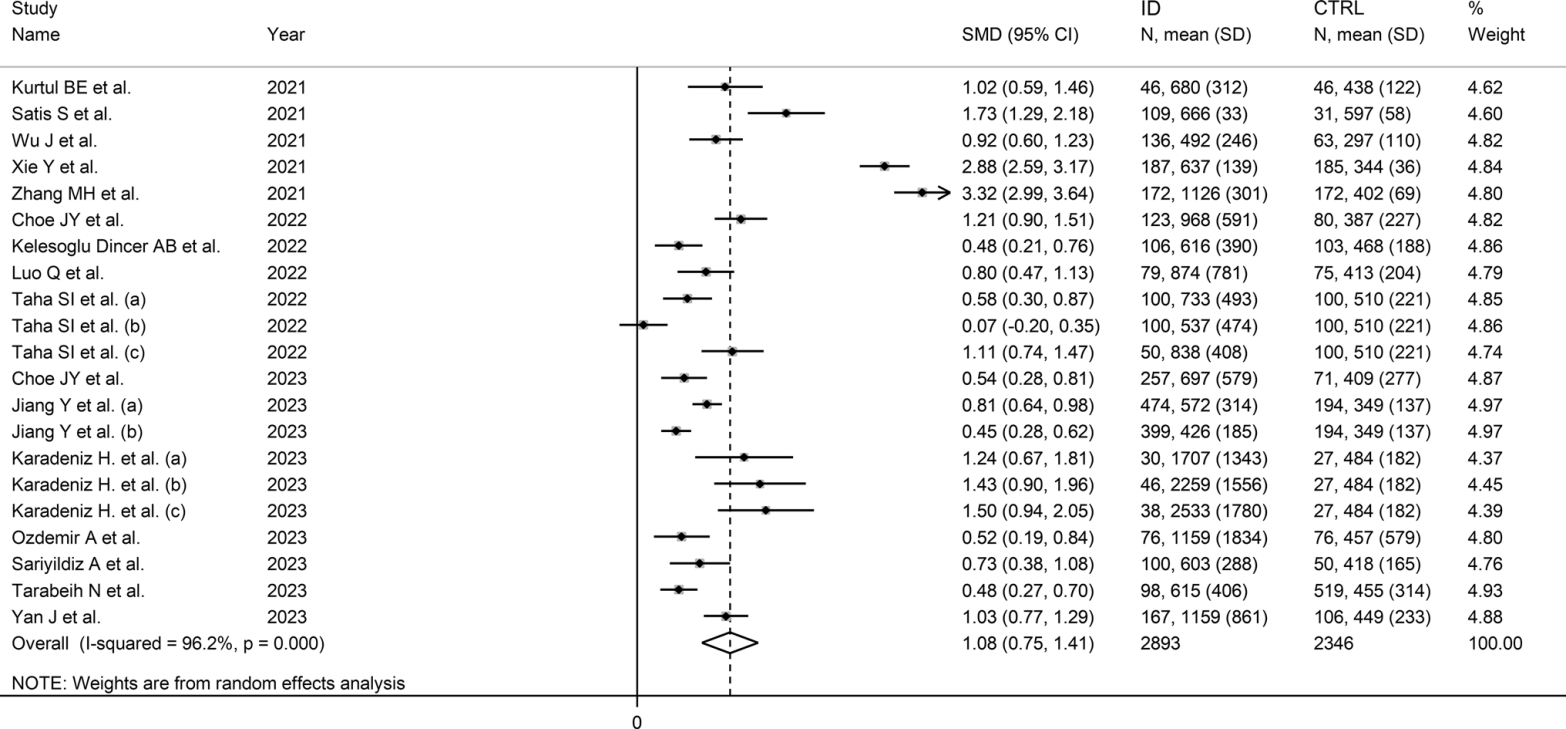
Forest plot of studies investigating the systemic inflammation index (SII) in patients with immunological diseases (IDs) and healthy controls

**Fig. 3:**
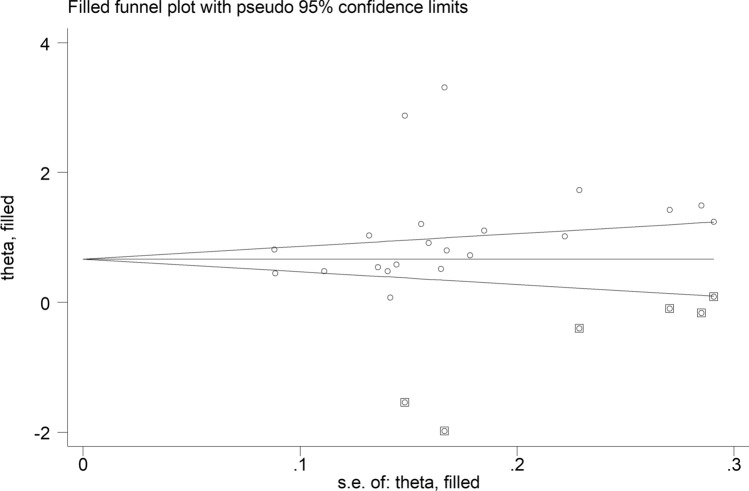
Funnel plot of studies investigating the association between the systemic inflammation index (SII) and immunological diseases (IDs) after “trimming-and-filling”. The circles enclosed by square and conventional circles represent dummy and genuine studies, respectively

The forest plot showed that the SII values were significantly higher in patients with IDs when compared with controls (SMD = 1.08, 95% CI 0.75 to 1.41, *p* < 0.001; I^2^ = 96.2%, *p* < 0.001; Fig. 2). The pooled SMD values were stable in sensitivity analysis, ranging between 0.96 and 1.13 (Supplementary Fig. 1). The Begg’s (*p* = 0.005), but not the Egger’s (*p* = 0.11), test indicated the presence of publication bias. The use of the “trim-and-fill” method led to the identification of six missing studies to be added to the left side of the funnel plot to ensure symmetry (Fig. 3). The resulting effect size was attenuated yet still significant (SMD = 0.70, 95% CI 0.31 to 1.08, *p* < 0.001).

**Fig. 4:**
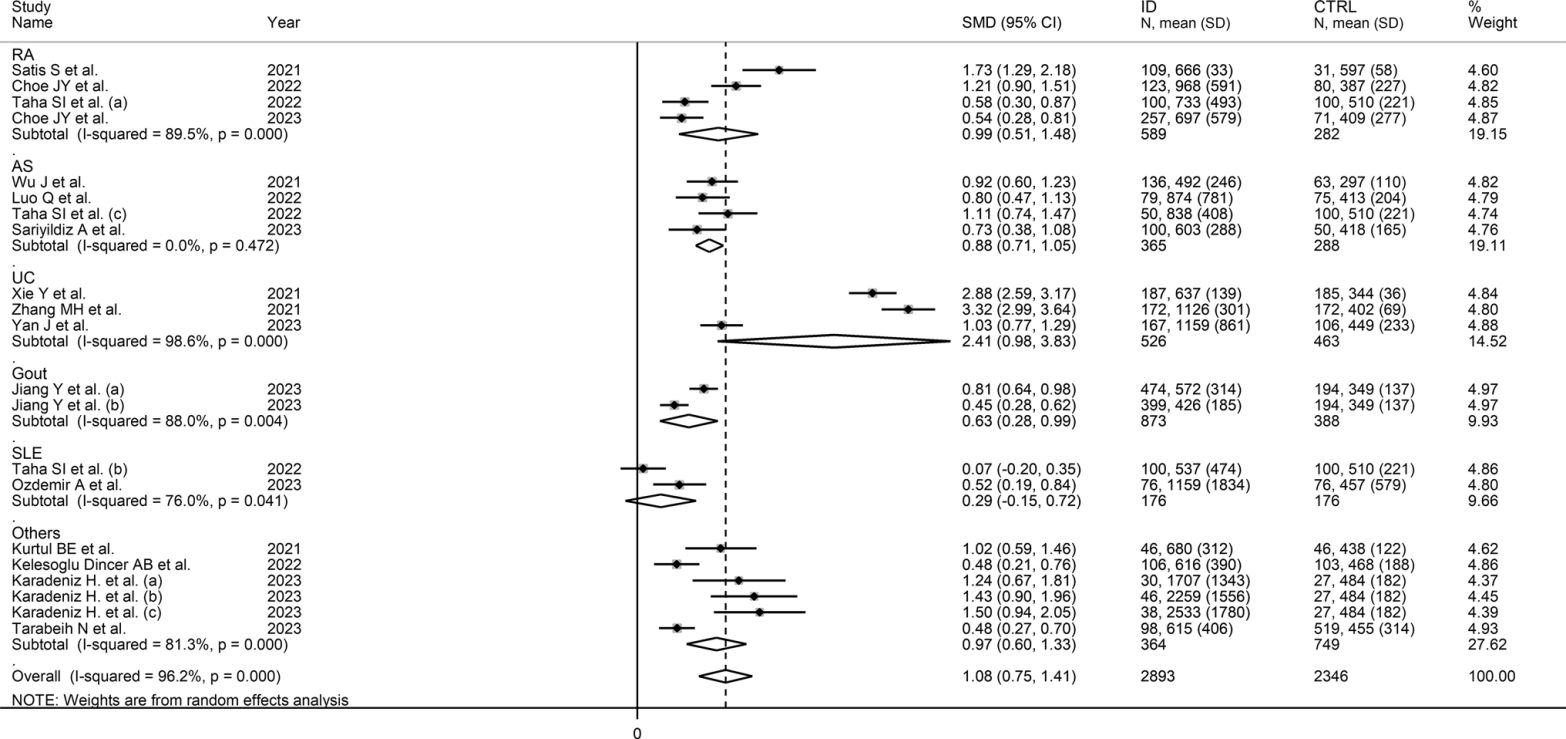
Forest plot of studies investigating the systemic inflammation index (SII) in patients with immunological diseases (IDs) and healthy controls according to type of ID

Univariate meta-regression analysis did not show any significant associations between the effect size and age (t = 1.02, *p* = 0.32), male to female ratio (t = 0.46, *p* = 0.65), sample size (t = − 0.27, *p* = 0.79), ID duration (*t* = − 0.83, *p* = 0.43), CRP (t = − 0.79, *p* = 0.44), or ESR (t = − 0.73, *p* = 0.48). By contrast, there was a significant inverse association with the year of publication (t = − 2.62, *p* = 0.017; Supplementary Fig. 2A and B). In subgroup analysis, the pooled SMD was significantly higher in studies in RA (SMD = 0.99, 95% CI 0.51–1.48, *p* < 0.001; I^2^ = 89.5%, *p* < 0.001), AS (SMD = 0.88, 95% CI 0.71–1.05, *p* < 0.001; I^2^ = 0.0%, *p* = 0.472), UC (SMD = 2.41, 95% CI 0.98–3.83, *p* = 0.001; I^2^ = 98.6%, *p* < 0.001) and gout (SMD = 0.63, 95% CI 0.28–0.99 *p* < 0.001; I^2^ = 88.0%, *p* = 0.004), but not SLE (SMD = − 0.29, 95% CI − 0.15–0.72, *p* = 0.20; I^2^ = 76.0%, *p* = 0.041), with a virtual absence of heterogeneity in the AS subgroup (Fig. 4). A non-significant trend (*p* = 0.07) toward a progressive reduction in the effect size was observed between studies conducted in China (SMD = 1.45, 95% CI 0.70–2.20, *p* < 0.001; I^2^ = 98.5%, *p* < 0.001), Turkey (SMD = 1.05, 95% CI 0.71–1.39, *p* < 0.001; I^2^ = 81.7%, *p* < 0.001), South Korea (SMD = 0.87, 95% CI 0.22–1.52, *p* = 0.008; I^2^ = 90.2%, *p* < 0.001), and Egypt (SMD = 0.58, 95% CI 0.02–1.14 *p* = 0.043; I^2^ = 90.1%, *p* = 0.004; Supplementary Fig. 3). There were non-significant (*p* = 0.16) differences in the pooled effect size between retrospective (SMD = 1.24, 95% CI 0.74–1.73, *p* < 0.001; I^2^ = 97.5%, *p* < 0.001) and prospective studies (SMD = 0.83, 95% CI 0.55–1.11, *p* < 0.001; I^2^ = 83.1%, *p* < 0.001; Supplementary Fig. 4).

**Fig. 5:**
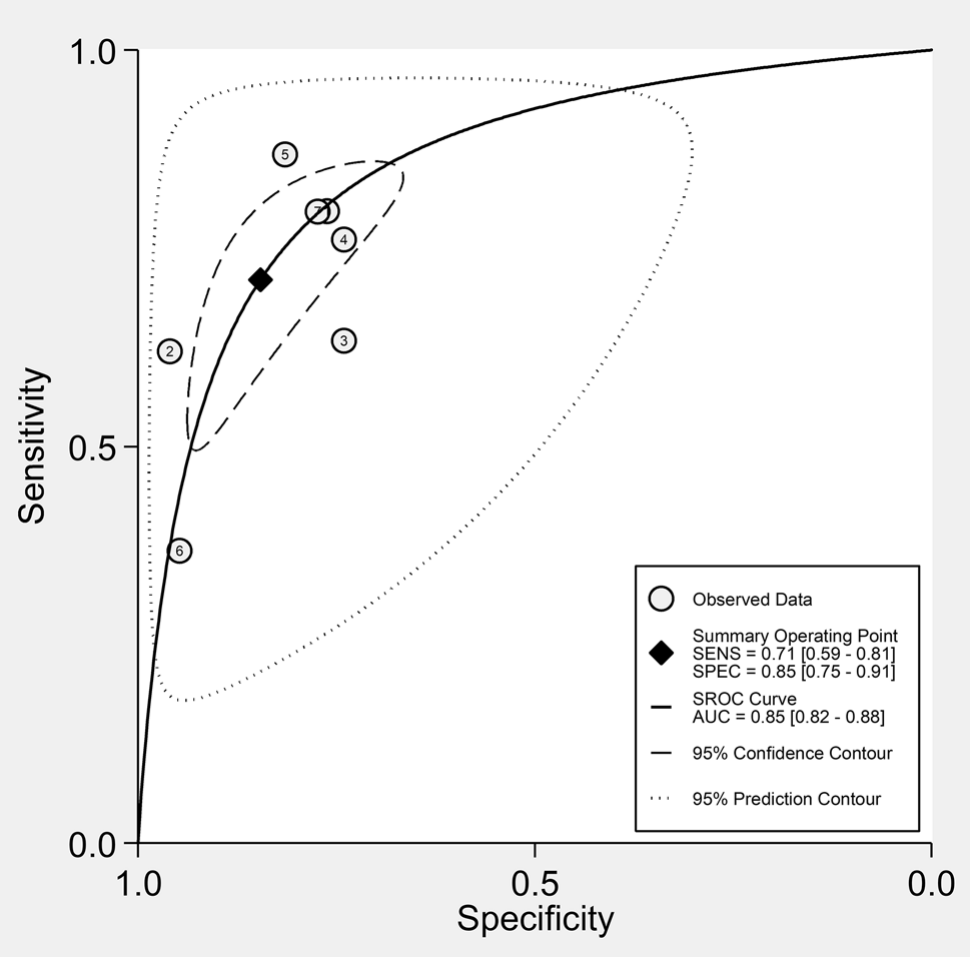
Summary receiving characteristics (SROC) curve with 95% confidence region and prediction region of the systemic inflammation index (SII) for the presence of immunological diseases (IDs)

Five studies reporting seven group comparisons investigated the diagnostic performance of the SII for the presence of IDs (Table 3) [46, 49, 53, 54, 57]. The pooled AUC value was 0.85 (95% CI 0.82–0.88) with the summary operating point at sensitivity of 71% (95% CI 59–81%) and specificity of 85% (95% CI 75–91%; Fig. 5).

**Table 3:** Studies investigating the diagnostic accuracy of the systemic inflammation index for immunological diseases

Study	Study design	n	AUC (95% CI)	Cut-off	Sensitivity (%)	Specificity (%)	Disease
Zhang MH et al. [46]	R	344	0.865 (0.814–0.891)	562.22	0.797	0.762	UC
Luo Q et al. [49]	P	154	0.832 (NR)	NR	0.622	0.96	AS
Karadeniz H. et al. (a), [53]	P	57	NR (NR)	535	0.633	0.741	IgG4-RD
Karadeniz H. et al. (b), [53]	P	73	NR (NR)	537	0.761	0.741	Sarcoidosis
Karadeniz H. et al. (c), [53]	P	65	NR (NR)	718	0.864	0.815	GPA
Ozdemir A et al. [54]	R	152	0.626 (0.540–0.707)	761	0.362	0.942	SLE
Yan J et al. [57]	R	273	0.861 (0.818–0.904)	619.1	0.7964	0.7736	UC

*AUC* area under the curve, *AS* ankylosing spondylitis, *CI* confidence interval, *GPA* granulomatosis polyangiitis, *IgG4-RD* IgG4-related disease, *NR* not reported, *P* prospective, *R* retrospective, *SLE* systemic lupus erythematosus, *UC* ulcerative colitis

The overall level of certainty was upgraded to moderate (rating 3) after considering the low-moderate risk of bias in all studies (no change), the high but partly explainable heterogeneity (no change), the lack of indirectness (no change), the relatively large effect size (SMD = 1.08, upgrade one level) [31], and the presence of publication bias which was addressed with the “trim-and-fill” method (no change).

### SII in patients with active disease and remission

We identified nine studies reporting 11 group comparisons which investigated a total of 2003 patients with IDs, 1261 with active disease and 742 in remission (mean age 46 years, 29% females) [43–45, 48, 50, 52, 54, 55, 57] (Table 4). Four studies were conducted in China [44, 45, 52, 57], four in Turkey [43, 48, 54, 55], and the remaining one in Egypt [50]. Three group comparisons investigated patients with AS [44, 50, 55], two with RA [43, 50], two with UC [45, 57], two with SLE [50, 54], one with gout [52], and one with PsA [48]. Seven studies were retrospective [43–45, 48, 52, 54, 57], and two prospective [50, 55]. The risk of bias was low in six studies [44, 45, 50, 52, 54, 55] and moderate in the remaining three [43, 48, 57] (Table 2).

**Table 4:** Studies investigating the systemic inflammation index in patients with active disease and remission

Study	Remission				Active disease				Disease type	Study design
	n	Age (years)	M/F	SII (mean ± SD)	n	Age (years)	M/F	SII (mean ± SD)		
Satis S et al. [43]	22	50	6/16	575 ± 34	87	50	18/69	702 ± 40	RA	R
Wu J et al. [44]	76	33	64/12	378 ± 129	60	34	54/6	697 ± 250	AS	R
Xie Y et al. [45]	36	NR	NR	397 ± 48	151	NR	NR	831 ± 262	UC	R
Kelesoglu Dincer AB et al. [48]	73	49	29/44	662 ± 317	32	47	18/14	1025 ± 526	PsA	R
Taha SI et al. (a), [50]	13	NR	NR	936 ± 797	87	NR	NR	720 ± 538	RA	P
Taha SI et al. (b), [50]	18	NR	NR	562 ± 607	82	NR	NR	556 ± 597	SLE	P
Taha SI et al. (c), [50]	15	NR	NR	710 ± 279	35	NR	NR	941 ± 641	AS	P
Jiang Y et al. [52]	399	42	399/0	426 ± 185	474	43	474/0	572 ± 314	Gout	R
Ozdemir A et al. [54]	22	NR	NR	1200 ± 2162	54	NR	NR	1027 ± 2250	SLE	R
Sariyildiz A et al. [55]	41	NR	NR	526 ± 221	59	NR	NR	591 ± 339	AS	P
Yan J et al. [57]	27	NR	NR	166 ± 44	140	NR	NR	232 ± 137	UC	R

*AS* ankylosing spondylitis, *M/F* male to female ratio, *NR* not reported, *OA* osteoarthritis, *P* prospective, *PsA* psoriatic arthritis, *R* retrospective, *RA* rheumatoid arthritis, *SII* systemic inflammation index, *SD* standard deviation, *SLE* systemic lupus erythematosus, *UC* ulcerative colitis

**Fig. 6:**
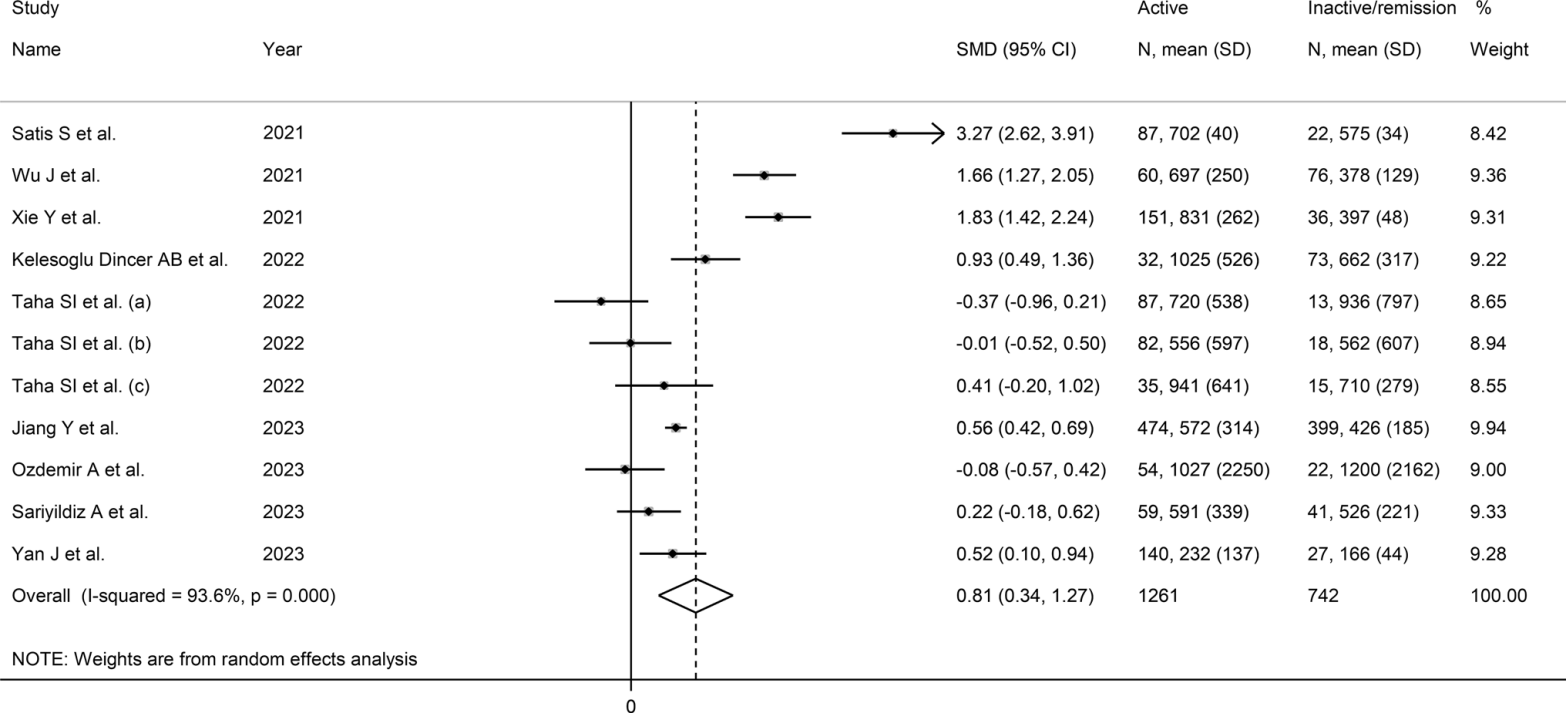
Forest plot of studies investigating the systemic inflammation index (SII) in patients with immunological diseases (IDs) with active disease and remission

**Fig. 7:**
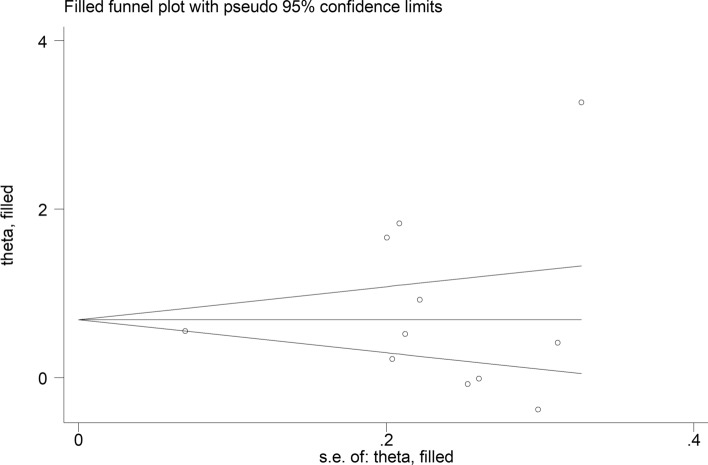
Funnel plot of studies investigating the association between the systemic inflammation index (SII) and active disease in patients with immunological diseases (IDs) after “trimming-and-filling”. The circles enclosed by square and conventional circles represent dummy and genuine studies, respectively

The forest plot showed that ID patients with active disease had significantly higher SII values when compared to those in remission (SMD = 0.81, 95% CI 0.34–1.27, *p* < 0.001; I^2^ = 93.6%, *p* < 0.001; Fig. 6). Sensitivity analysis showed stability of the pooled SMD values (effect size range between 0.58 and 0.92; Supplementary Fig. 5). There was no evidence of publication bias according to either the Begg’s (*p* = 1.00) or the Egger’s test (*p* = 0.56). No missing study was identified using the “trim-and-fill” method (Fig. 7).

**Fig. 8:**
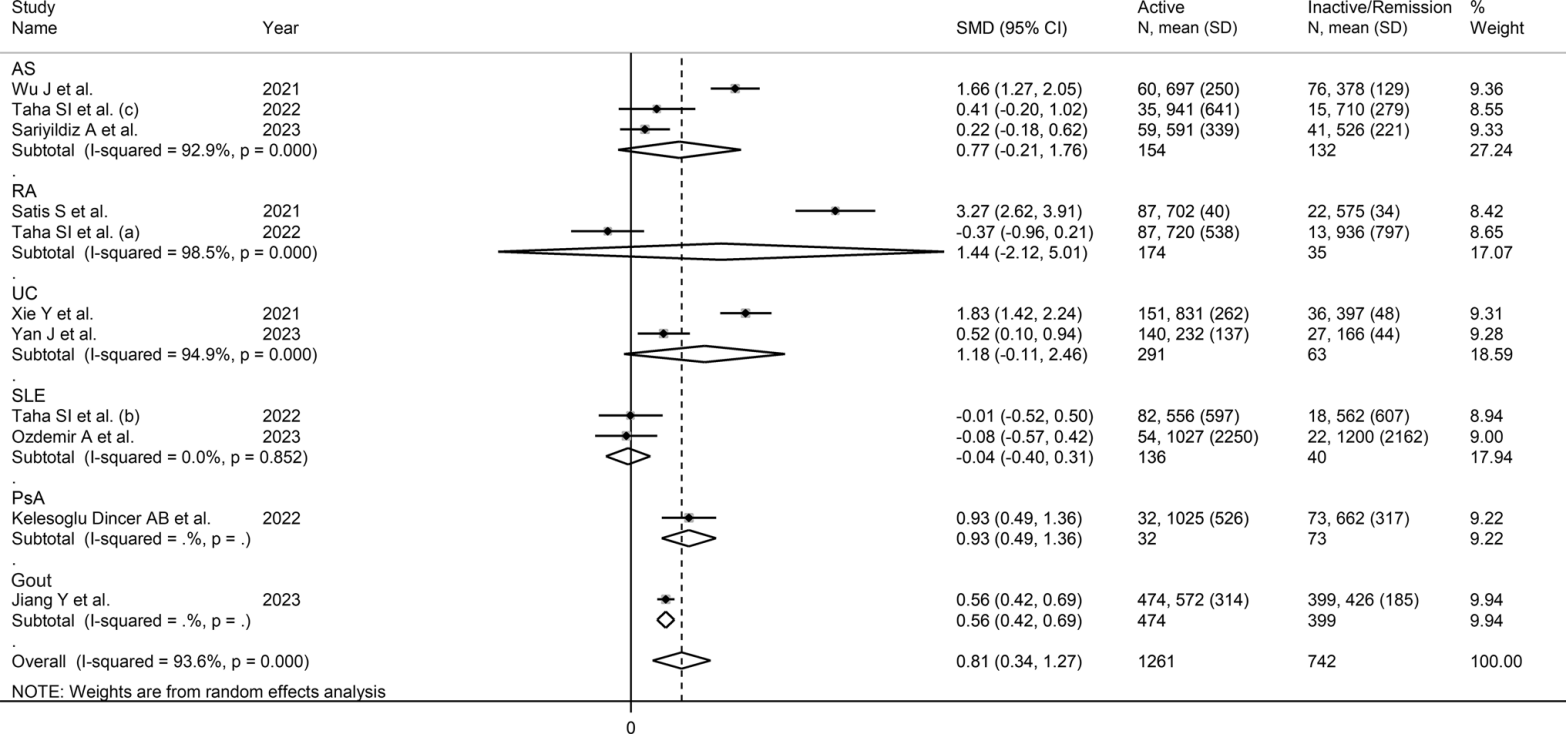
Forest plot of studies investigating the systemic inflammation index (SII) in patients with immunological diseases (IDs) with active disease and remission according to type of ID

There were non-significant associations between the effect size and age (t = − 0.88, *p* = 0.40), male to female ratio (t = 0.74, *p* = 0.48), sample size (t = -0.05, *p* = 0.96), CRP (t = − 1.96, *p* = 0.09), or ESR (t = − 1.76, *p* = 0.12) in univariate meta-regression analysis. By contrast, a significant inverse association was observed with the year of publication (t = − 3.09, *p* = 0.013; Supplementary Fig. 6A and B). In subgroup analysis, the pooled SMD was similar between patients with AS (SMD = 0.77, 95% CI − 0.21–1.76, *p* = 0.12; I^2^ = 92.9%, *p* < 0.001), RA (SMD = 1.44, 95% CI − 2.12–5.01, *p* = 0.42; I^2^ = 98.5%, *p* < 0.001), UC (SMD = 1.18, 95% CI − 0.11–2.46, *p* = 0.07; I^2^ = 94.9%, *p* < 0.001) and SLE (SMD = − 0.04, 95% CI − 0.40–0.31, *p* = 0.81; I^2^ = 0.0%, *p* = 0.852) with a virtually absent heterogeneity in the SLE subgroup (Fig. 8). The pooled SMD was statistically significant in studies conducted in China (SMD = 1.13, 95% CI 0.44–1.82, *p* = 0.001; I^2^ = 94.7%, *p* < 0.001), but not Turkey (SMD = 1.06, 95% CI − 0.15–2.28, *p* = 0.09; I^2^ = 96.1%, *p* < 0.001) or Egypt (SMD = 0.00, 95% CI − 0.44–0.42, *p* = 0.99; I^2^ = 40.0%, *p* = 0.19), with a relatively lower heterogeneity in the latter subgroup (Supplementary Fig. 7). Furthermore, the effect size was statistically significant in retrospective (SMD = 1.22, 95% CI 0.59–1.84, *p* < 0.001; I^2^ = 95.3%, *p* < 0.001) but not in prospective studies (SMD = 0.08, 95% CI − 0.22–0.38, *p* = 0.61; I^2^ = 25.8%, *p* = 0.257; Supplementary Fig. 8).

**Fig. 9:**
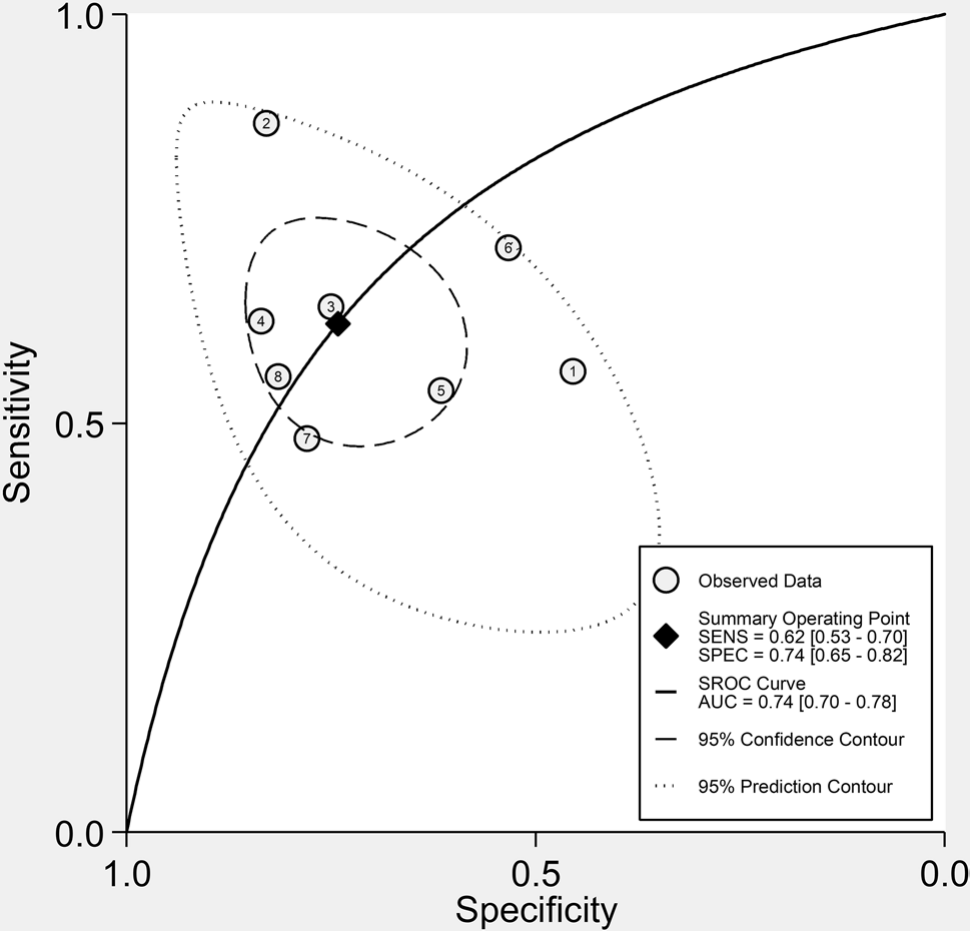
Summary receiving characteristics (SROC) curve with 95% confidence region and prediction region of the systemic inflammation index (SII) for the presence of active disease in patients with immunological diseases (IDs)

Seven studies with eight group comparisons investigated the diagnostic performance of the SII for active disease [43–45, 48, 50, 52, 57] (Table 5). The pooled sensitivity, specificity, and AUC values of the SROC were 62% (95% CI 53–70%), 74% (95% CI 65–82%), and 0.74 (95% CI 0.70–0.78), respectively (Fig. 9).

**Table 5:** Studies investigating the diagnostic accuracy of the systemic inflammation index for disease activity

Study	Study design	n	AUC (95% CI)	Cut-off	Sensitivity (%)	Specificity (%)	Disease
Satis S et al [43]	R	109	0.643 (0.534–0.753)	574.2	0.563	0.455	RA
Wu J et al [44]	R	136	0.877 (0.813–0.941)	513.2	0.868	0.833	AS
Xie Y et al. [45]	R	187	0.711 (0.630–0.791)	485.95	0.641	0.75	UC
Kelesoglu Dincer AB et al. [48]	R	105	0.753 (0.650–0.855)	800	0.625	0.836	PsA
Taha SI et al. (a), [50]	P	100	0.622 (0.449–0.794)	691.55	0.54	0.615	RA
Taha SI et al. (b), [50]	P	100	0.674 (0.504–0.845)	697.66	0.714	0.533	SLE
Jiang Y et al. [52]	R	873	0.647 (0.610–0.683)	568.5	0.481	0.779	Gout
Yan J et al. [57]	R	167	0.691 (0.588–0.974)	1068	0.5571	0.8148	UC

*AS* ankylosing spondylitis, *AUC* area under the curve, *CI* confidence interval, *OA* osteoarthritis, *P* prospective, *PsA* psoriatic arthritis, *R* retrospective, *RA* rheumatoid arthritis, *SLE* systemic lupus erythematosus, *UC* ulcerative colitis

The overall level of certainty was upgraded to moderate (rating 3) after considering the low-moderate risk of bias in all studies (no change), the high but partly explainable heterogeneity (no change), the lack of indirectness (no change), the relatively large effect size (SMD = 0.81, upgrade one level) [31], and the absence of publication bias (no change).

## Discussion

The significant differences in the SII between IDs patients and healthy controls and between IDs patients with active disease and remissions reported in this systematic review and meta-analysis suggests the potential clinical utility of the SII as a diagnostic biomarker of IDs. The capacity of the SII to discriminate between different groups was considered excellent for the presence of IDs (pooled AUC = 0.85) and acceptable for the presence of active disease (pooled AUC = 0.74) [58, 59]. Sensitivity analyses confirmed the stability of the results of the meta-analysis. In meta-regression, the effect size was not significantly associated with several demographic and clinical characteristics, particularly ID duration and conventional biomarkers of inflammation (CRP and ESR). This suggests that the between-group differences in the SII a) are also present in the early phases of the disease and b) may provide clinical information that complements or enhances that provided by available biomarkers of inflammation. Interestingly, subgroup analysis identified differences in the effect size between different types of IDs for the presence of IDs but not for the presence of active disease in patients with IDs.

The SII was initially studied in patients with liver cancer [60], with subsequent investigations reporting significant associations with clinical outcomes in different types of cancer [25, 61–63], as well as in other disease states [26–28]. Studies conducted in patients with atherosclerosis have also reported the potential prognostic superiority of the SII over conventional risk factors [64]. Furthermore, in patients with COVID-19 the SII, but not other hematological indices such as the aggregate index of systemic inflammation, the neutrophil-to-lymphocyte ratio, the monocyte-to-lymphocyte ratio, the platelet-to-lymphocyte ratio, and the systemic inflammation response index, was independently associated with adverse outcomes [29]. The potential diagnostic superiority of the SII specifically in IDs is further supported by the results of studies investigating the diagnostic performance of the CRP and the ESR in primary care using datalink sources. For example, a study identified a total of 160,000 patients from the Clinical Practice Research Datalink in the UK who had conventional inflammatory markers tested in 2014 [15, 65]. The primary outcome was defined as any autoimmune disease or cancer coded within one year, or infection coded within one month of the index date of inflammatory marker testing. In the final cohort of 136,691 patients (median age of 55.4 years, 62% female), the AUC for autoimmune conditions was 0.71 (95% CI 0.60–0.72) for the CRP and 0.71 (95% CI 0.69–0.72) for the ESR [15]. These values are considerably lower than the pooled AUC values observed in our study for the diagnosis of IDs (0.85, 95% CI 0.82–0.88). Despite these promising findings, appropriately designed prospective studies are warranted to investigate the diagnostic and prognostic capacity of the SII, singly or in combination with other biomarkers of inflammation and/or clinical parameters, in patients with different types of ID.

Our study has several strengths, including the assessment of the SII in different types of IDs within the autoinflammatory-autoimmune continuum including autoinflammatory, mixed-pattern, and autoimmune diseases [1, 7, 8], the assessment of possible associations between the effect size and several study and patient characteristics, and a rigorous evaluation of the risk of bias and the certainty of evidence. Furthermore, sensitivity analysis ruled out the effect of individual studies on the overall effect size. Important limitations include the focus of the studies identified in our search on a restricted number of IDs (RA, AS, UC, gout, SLE, PsA, OA, uveitis, sarcoidosis, GPA, and IgG4-RD), and the lack of evidence from studies in specific geographical location, particularly Europe and North and South America. These issues require further study given the established evidence of differences in inflammatory response across different types of IDs and ethnic groups [66–71].

In conclusion, our systematic review and meta-analysis has shown the potential utility of the SII in diagnosing the presence of IDs and active disease. However, additional research is required to confirm these observations and determine whether this haematologically derived index can enhance the diagnostic capacity of current biomarkers and other clinical parameters in patients with different types of IDs and ethnicity.

## Supplementary Information

Below is the link to the electronic supplementary material.

Supplementary file 1 (TIF 4828 KB)

Supplementary file 2 (TIF 5351 KB)

Supplementary file 3 (TIF 4310 KB)

Supplementary file 4 (TIF 4334 KB)

Supplementary file 5 (TIF 3514 KB)

Supplementary file 6 (TIF 4289 KB)

Supplementary file 7 (TIF 3643 KB)

Supplementary file 8 (TIF 3515 KB)

Supplementary file 9 (DOCX 13 KB)

Supplementary file 10 (DOCX 20 KB)

Supplementary file 11 (DOCX 34 KB)

## Data Availability

The data that support the findings of this systematic review and meta-analysis are available from AZ upon reasonable request.
