# Supplementary figures and images for: The diagnostic role of the systemic inflammation index in patients with immunological diseases: a systematic review and meta-analysis
Source: Clin Exp Med. 2024 Jan 29;24(1):27. doi: 10.1007/s10238-024-01294-3 (PMC10824868; doi:10.1007/s10238-024-01294-3)

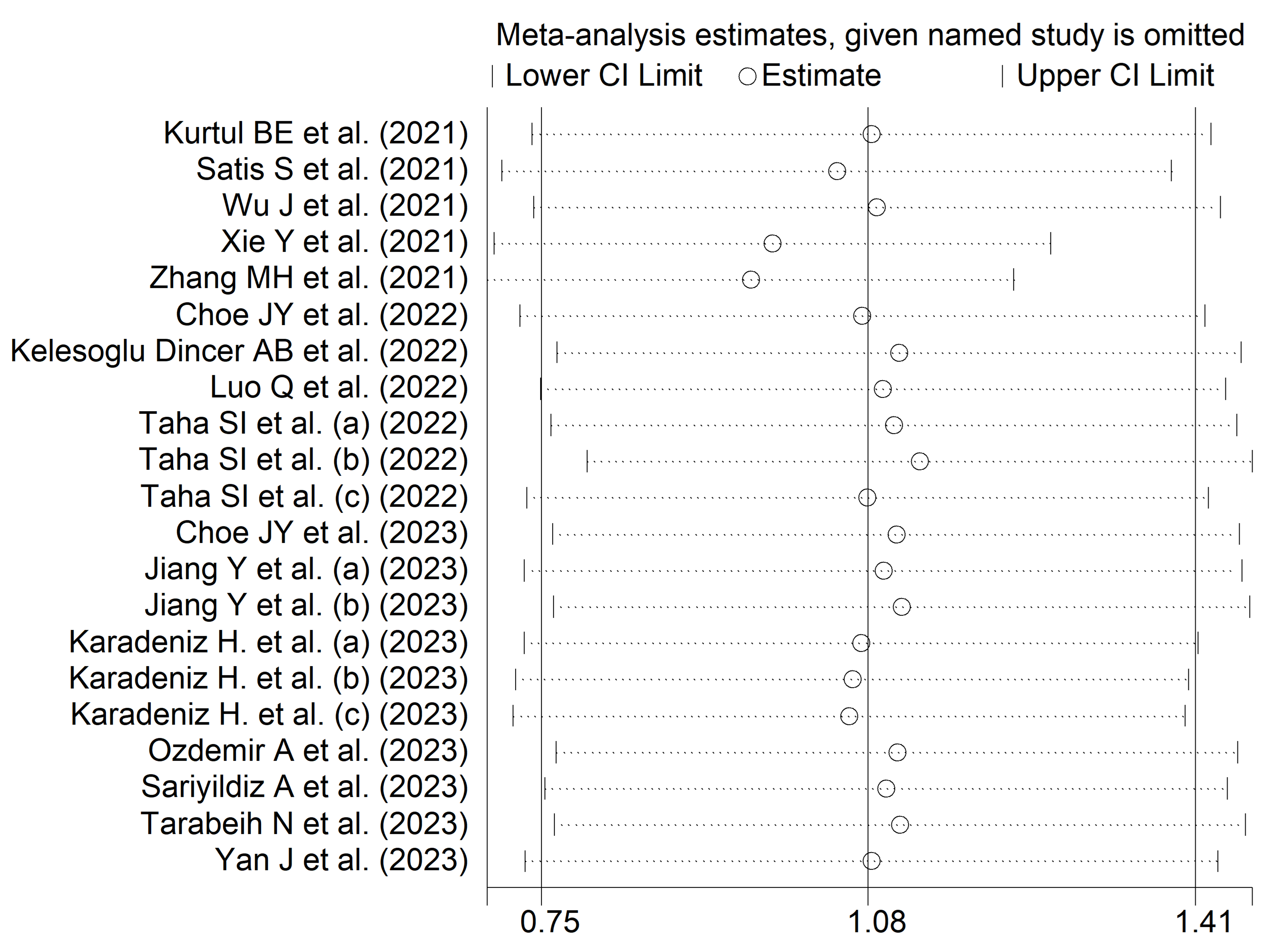

Supplement: Supplementary file 1 — (TIF 4828 KB) [file 10238_2024_1294_MOESM1_ESM.tif]

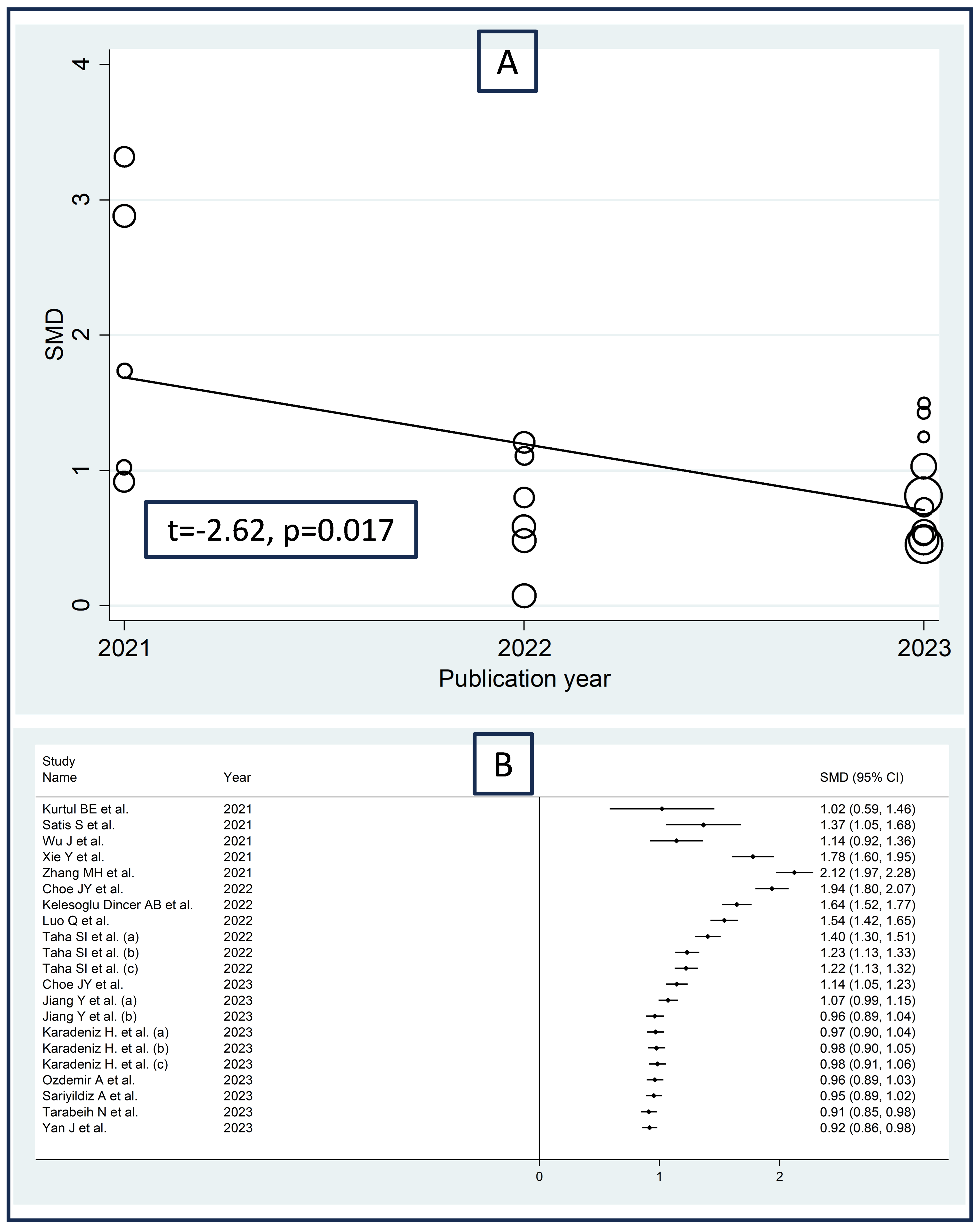

Supplement: Supplementary file 2 — (TIF 5351 KB) [file 10238_2024_1294_MOESM2_ESM.tif]

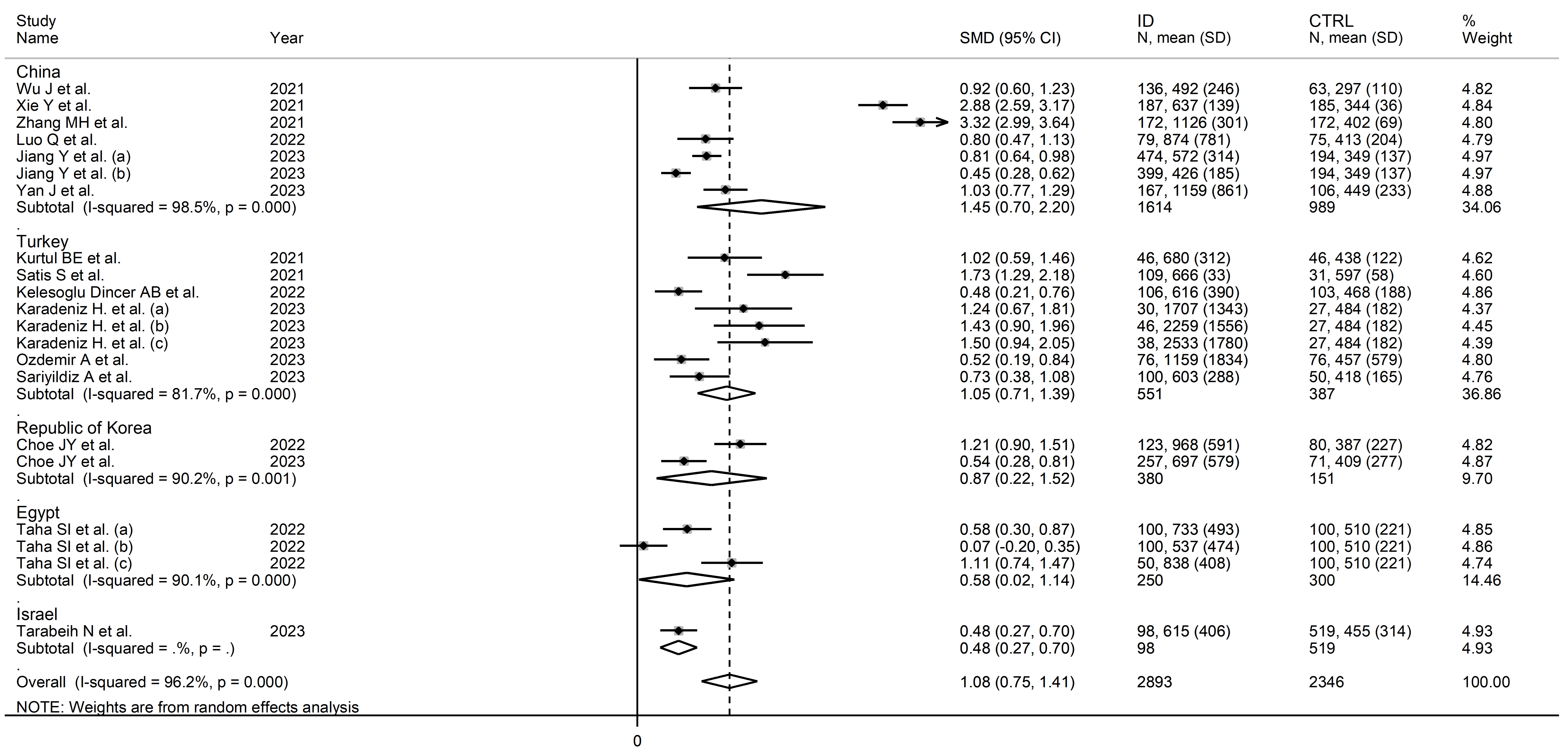

Supplement: Supplementary file 3 — (TIF 4310 KB) [file 10238_2024_1294_MOESM3_ESM.tif]

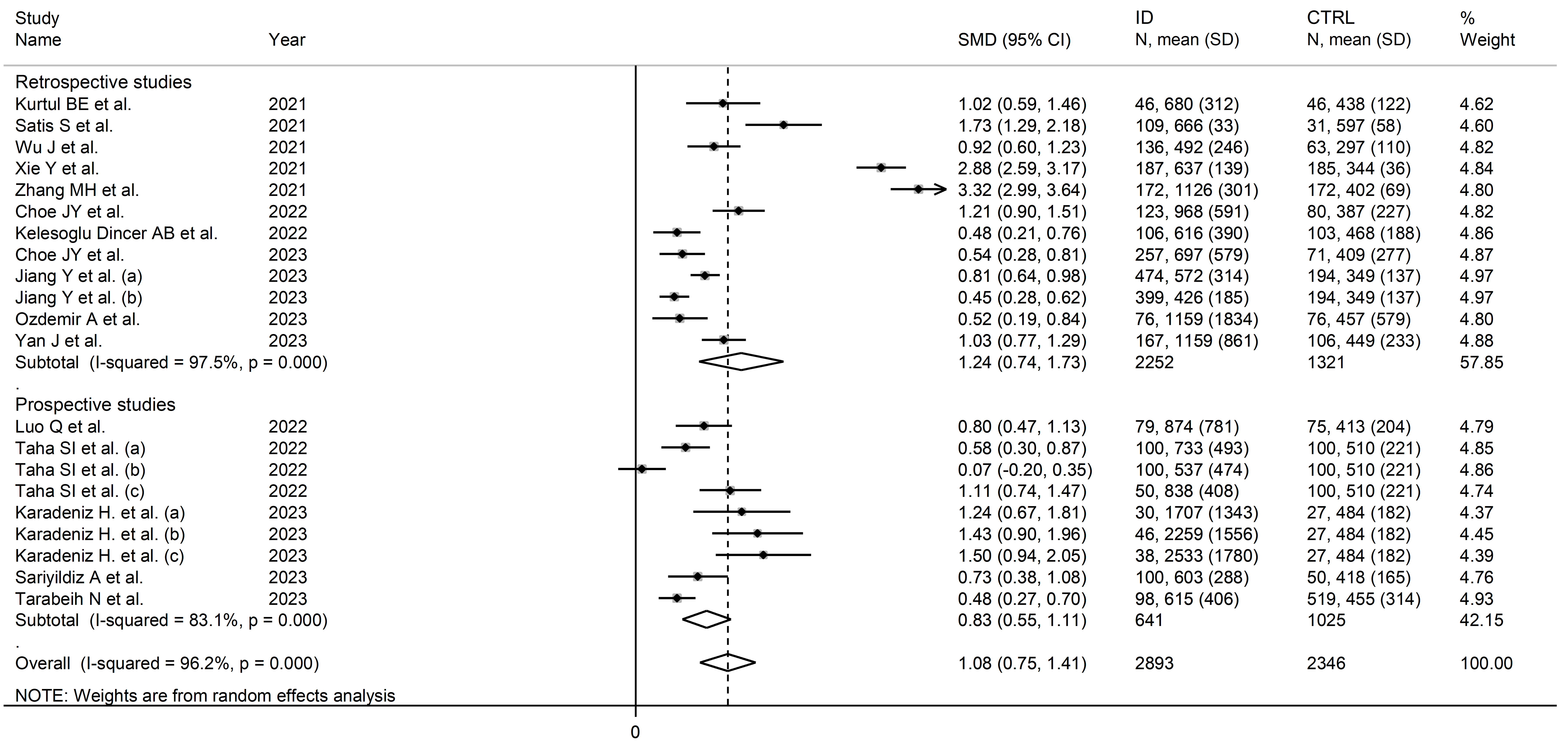

Supplement: Supplementary file 4 — (TIF 4334 KB) [file 10238_2024_1294_MOESM4_ESM.tif]

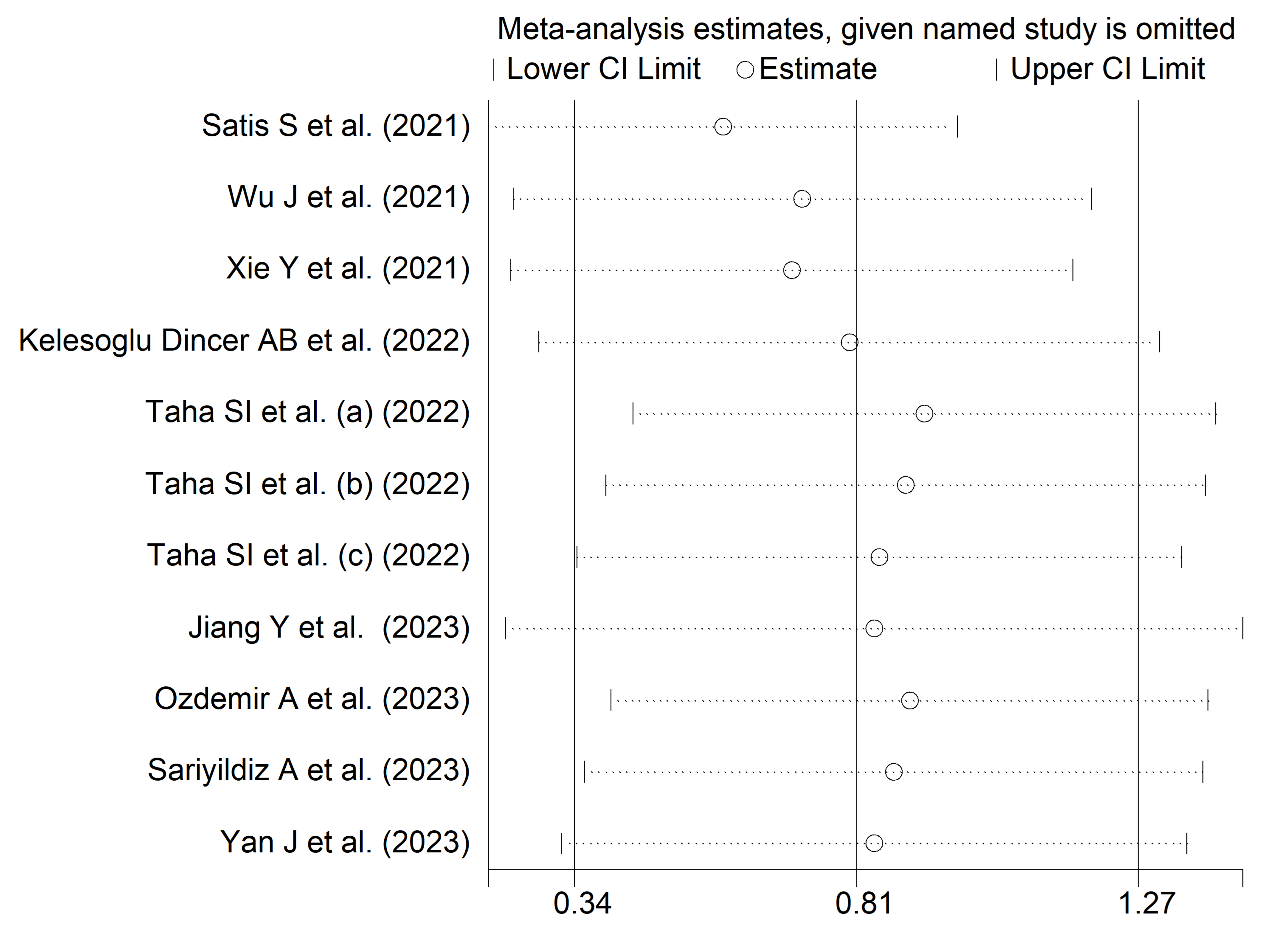

Supplement: Supplementary file 5 — (TIF 3514 KB) [file 10238_2024_1294_MOESM5_ESM.tif]

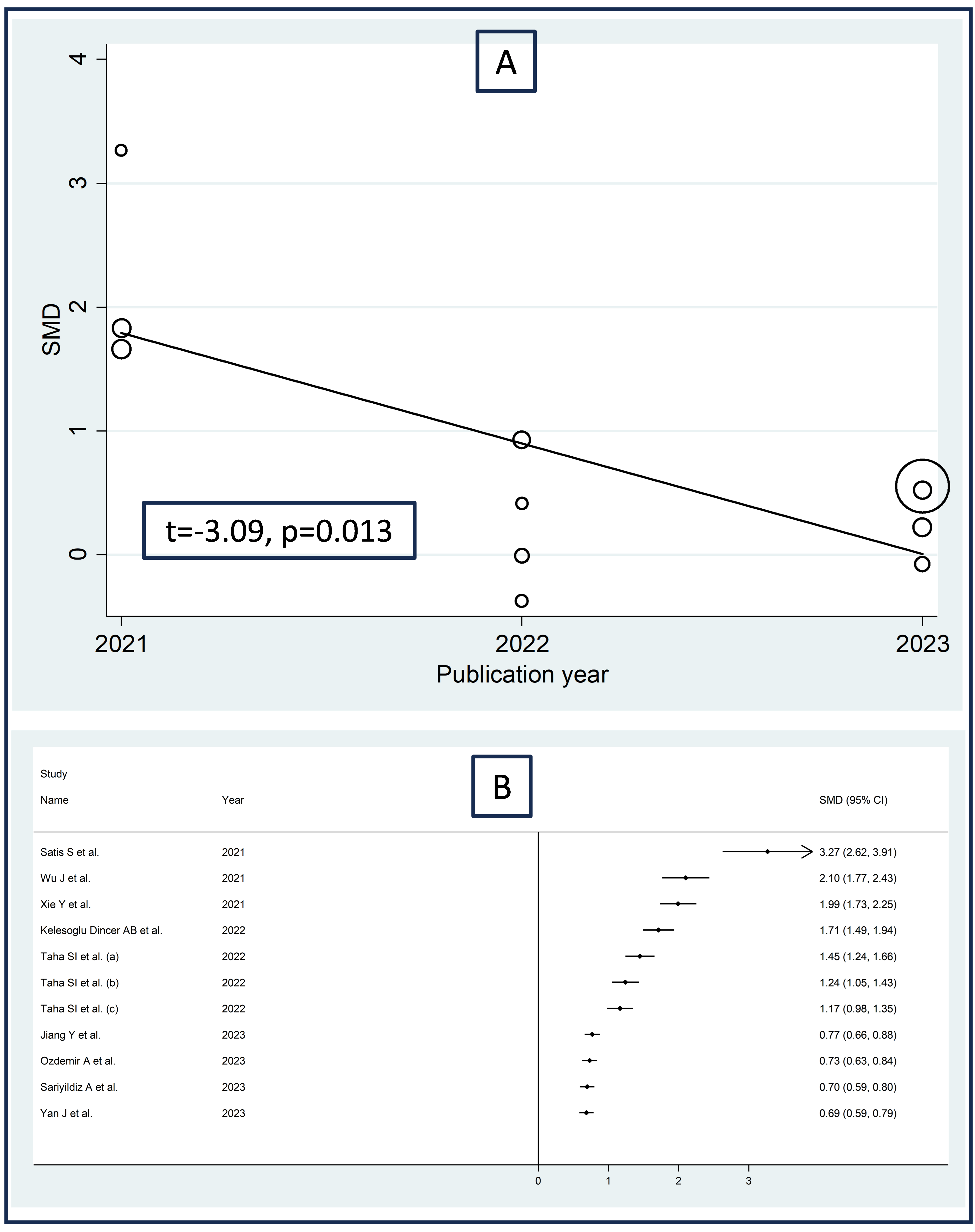

Supplement: Supplementary file 6 — (TIF 4289 KB) [file 10238_2024_1294_MOESM6_ESM.tif]

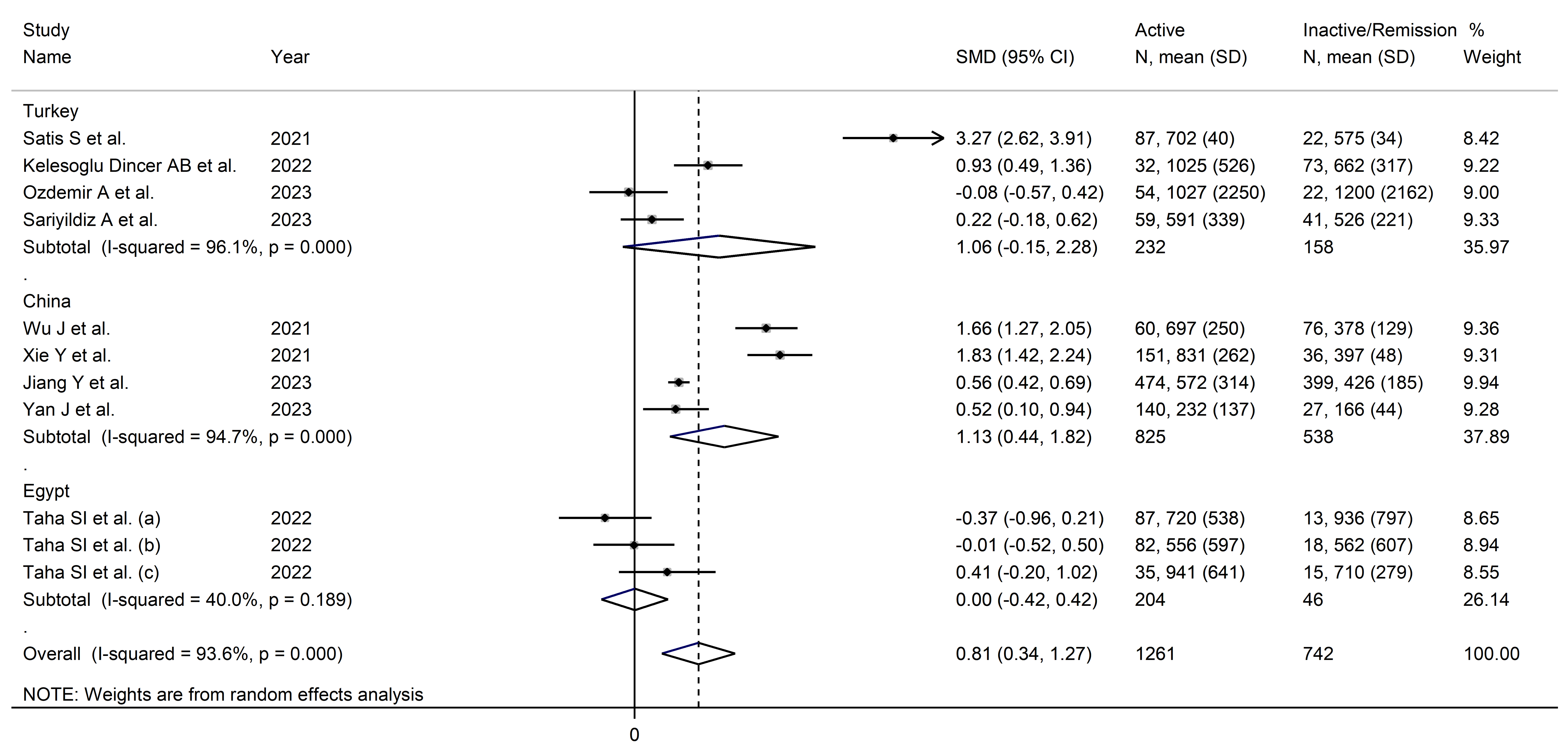

Supplement: Supplementary file 7 — (TIF 3643 KB) [file 10238_2024_1294_MOESM7_ESM.tif]

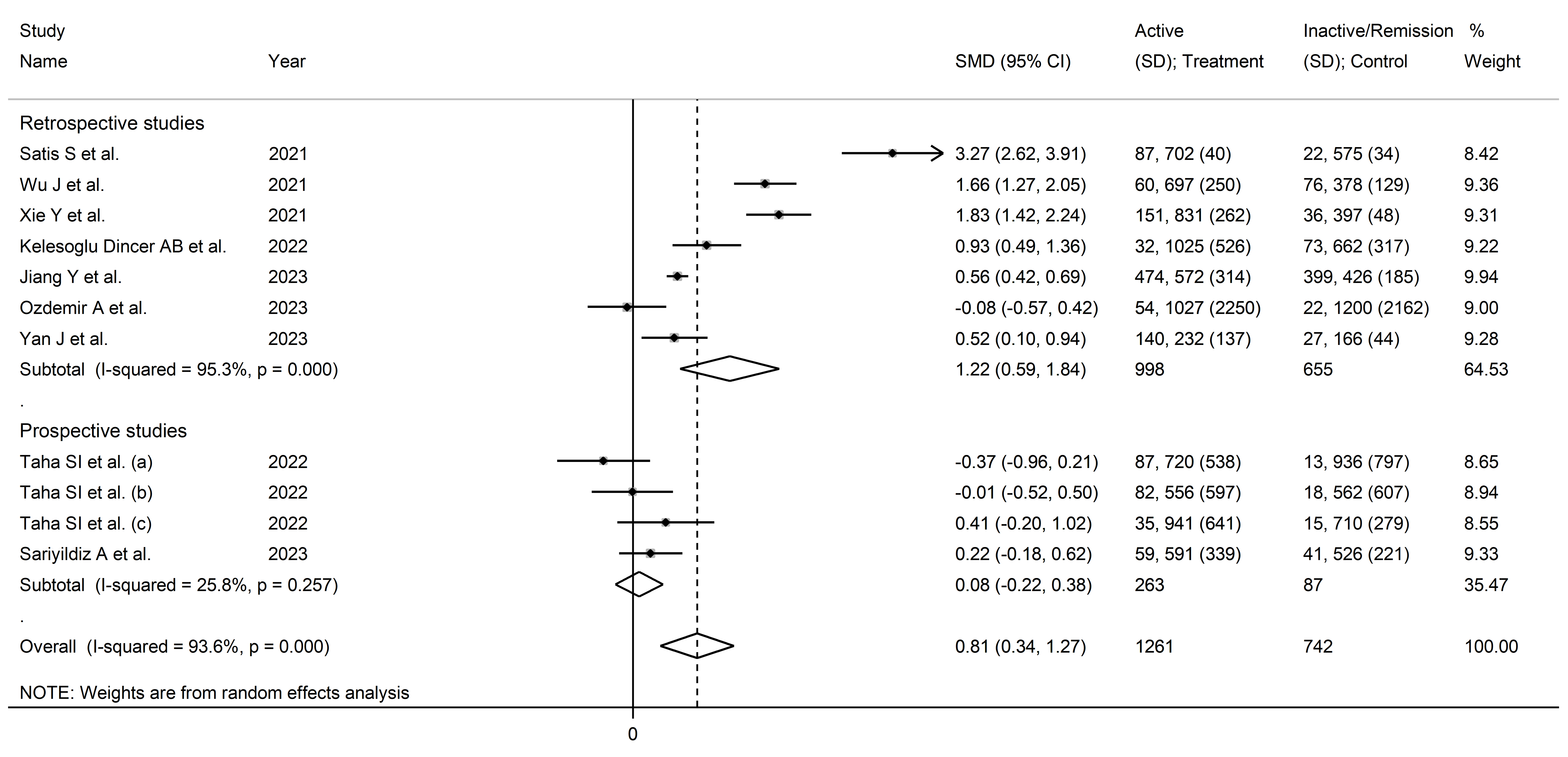

Supplement: Supplementary file 8 — (TIF 3515 KB) [file 10238_2024_1294_MOESM8_ESM.tif]
