# Supplementary material for: The diagnostic role of the systemic inflammation index in patients with immunological diseases: a systematic review and meta-analysis
Source: Clin Exp Med. 2024 Jan 29;24(1):27. doi: 10.1007/s10238-024-01294-3 (PMC10824868; doi:10.1007/s10238-024-01294-3)
Supplement: Supplementary file 9 — (DOCX 13 KB) [file 10238_2024_1294_MOESM9_ESM.docx]

**Supplementary figure legends**

**Supplementary Figure 1.** Sensitivity analysis of the association between the systemic inflammation index (SII) and immunological diseases (IDs).

**Supplementary Figure 2.** Bubble plot reporting the univariate meta-regression analysis between the effect the size of the difference between patients with immunological diseases (IDs) and healthy controls and publication year (A) and cumulative meta-analysis of the systemic inflammation index (SII) based on publication year (B).

**Supplementary Figure 3.** Forest plot of studies investigating the systemic inflammation index (SII) in patients with immunological diseases (IDs) and healthy controls according to the study country.

**Supplementary Figure 4.** Forest plot of studies investigating the systemic inflammation index (SII) in patients with immunological diseases (IDs) and healthy controls according to study design.

**Supplementary Figure 5.** Sensitivity analysis of the association between the systemic inflammation index (SII) and active disease in patients with immunological diseases (IDs).

**Supplementary Figure 6.** Bubble plot reporting the univariate meta-regression analysis between the effect the size of the difference between patients with immunological diseases (IDs) with active disease and remission and publication year (A) and cumulative meta-analysis of the systemic inflammation index (SII) based on publication year (B).

**Supplementary Figure 7.** Forest plot of studies investigating the systemic inflammation index (SII) in patients with immunological diseases (IDs) with active disease and remission according to study country.

**Supplementary Figure 8.** Forest plot of studies investigating the systemic inflammation index (SII) in patients with immunological diseases (IDs) with active disease and remission according to study design.
